# IDENTIFYING PATTERNS OF CHANGE IN SPIRITUAL WELL-BEING OVER TIME AMONG NEURO-ONCOLOGY CAREGIVERS

**DOI:** 10.1093/geroni/igad104.3407

**Published:** 2023-12-21

**Authors:** Jiayi Zhu, Paula Sherwood, Susan Sereika, Heidi Donovan

**Affiliations:** University of Pittsburgh, Pittsburgh, Pennsylvania, United States; University of Pittsburgh, Pittsburgh, Pennsylvania, United States; University of Pittsburgh, Pittsburgh, Pennsylvania, United States; University of Pittsburgh, Pittsburgh, Pennsylvania, United States

## Abstract

Spiritual well-being/spirituality has been recognized as a protective factor against caregiver burden, depression, anxiety, and poor quality of life in family caregivers (CG) of patients diagnosed with serious illnesses, such as cancer. However, inconsistent previous findings may be attributed to the limited understanding of spirituality as a dynamic, multidimensional concept. To address this gap, we conducted a secondary analysis using longitudinal data from two independent studies of CGs of patients with primary malignant brain tumors (pooled N=261) to identify trajectories in overall spirituality (FACIT-SP) and its 3 sub-dimensions (faith, meaning, peace) from diagnosis to 10-12 months post-diagnosis, as well as predictors and outcomes associated with trajectory group membership. For overall spirituality, 3 unique trajectories were identified: low-increasing, medium-stable, and high-stable. The faith and meaning sub-dimensions had 3 and 2 stable trajectories, respectively, while the peace sub-dimension showed low-increasing, medium-stable, and high-increasing trajectories. Older age and higher levels of perceived social support at baseline were associated with increased odds (up to 1.06 and 1.20, respectively) of being in the high-stable/high-increasing trajectories for overall spirituality and the sub-dimensions. The trajectories of overall spirituality and sub-dimensions were positively associated with CG’s mental well-being (η2=.030-.069) and self-esteem (η2=.072-.162), and negatively associated with CG’s depression (η2=.053-.319), anxiety (η2=.055-.271), and feeling of abandonment (η2=.032-.160) at 10-12 months. This study supports recognizing spirituality in CGs as a dynamic, multidimensional concept. Future interventions focusing on early screening in younger adults and supportive measures for low spiritual well-being through improving social support might improve important CG outcomes.

